# Real-Time Far-Field BCSDF Filtering

**DOI:** 10.3390/jimaging11050158

**Published:** 2025-05-16

**Authors:** Junjie Wei, Ying Song

**Affiliations:** School of Computer Science and Technology, Zhejiang Sci-Tech University, Hangzhou 310018, China; 202220601011@mails.zstu.edu.cn

**Keywords:** real-time rendering, LoD, tangent map filtering, von Mises–Fisher distribution

## Abstract

The real-time rendering of large-scale curve-based surfaces (e.g., hair, fabrics) requires efficient handling of bidirectional curve-scattering distribution functions (BCSDFs). While curve-based material models are essential for capturing anisotropic reflectance characteristics, conventional prefiltering techniques encounter challenges in jointly resolving micro-scale BCSDFs variations with tangent distribution functions (TDFs) at pixel-level accuracy. This paper presents a real-time BCSDF filtering framework that achieves high-fidelity rendering without precomputation. Our key insight lies in formulating each pixel’s scattering response as a mixture of von Mises–Fisher (vMF) distributions, enabling analytical convolution between micro-scale BCSDFs and TDFs. Furthermore, we derive closed-form expressions for the integral of TDF-BCSDF products, avoiding the need for numerical approximation and heavy precomputation. Our method demonstrates state-of-the-art performance, achieving results comparable to 1000 spp Monte Carlo simulations under parallax-free conditions, where it improves the mean squared error (MSE) by one to two orders of magnitude over baseline methods. Qualitative comparisons and error analysis confirm both visual fidelity and computational efficiency.

## 1. Introduction

Modern curve-based rendering techniques have progressed from simplified appearance models to sophisticated representations capable of encoding spatially varying reflectance and transmittance in complex microstructures, such as knitted fabrics. While the bidirectional curve-scattering distribution functions (BCSDFs) enable the physically accurate rendering of yarn- or fiber-level details, its computational complexity introduces substantial performance degradation. Conventional physics-based rendering pipelines exhibit prohibitive costs (minutes to hours per frame), fundamentally constraining real-time integration of BCSDFs.

Previous works [1,2] employ feature maps (e.g., normal/tangent maps) as parametric proxies for yarn or fiber geometry reconstruction, striking an optimal balance between fidelity and performance. While surface-based representations demonstrate superior efficiency in storage and rendering, they lack efficient filtering mechanisms during real-time filtering. This limitation stems from the need to compute convolutions over high-dimensional input signals [3], which typically demands Monte Carlo integration with thousands of samples per pixel. Although existing work has investigated prefiltering techniques for BRDFs [4] and normal maps [5,6,7,8], the joint convolution of BCSDFs with tangent maps remains unaddressed.

**Figure 1:**
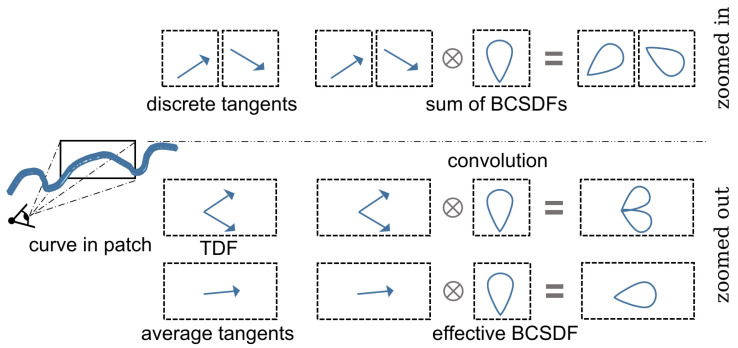
For high-magnification scenarios (first row), we model individual pixels as discrete tangents and render them via separate shading summation. At lower resolutions (second row), where a single patch spans multiple tangents, conventional linear averaging introduces artifacts, including curve linearization and shading inaccuracies. Instead, our approach preserves all shading characteristics through a TDF, obtaining an effective BCSDF via the convolution of the TDF with the BCSDF.

In large-scale rendering, a screen-space pixel footprints typically encompass numerous sub-pixel geometric details. The critical challenge for efficient filtering lies in computing an aggregated *effective BCSDF*, as shown in Figure 1, which statistically represents the collective behavior of micro-scale BCSDFs within the footprint. Existing approaches either neglect BCSDFs entirely or fail to provide closed-form solutions for this spatial aggregation problem.

In this paper, we propose an analytical framework for deriving closed-form effective BCSDFs. Specifically, we demonstrate a real-time far-field BCSDF filtering technique. Note that the term ’far-field’ characterizes the inherent scattering properties of BCSDFs, where macroscopic reflectance and transmission dominate over near-field fiber-level scattering interactions. Our method extends the three-lobe BCSDF model (reflection R, transmission TT, and diffuse D) by reparameterizing its components to enable analytical convolution with the tangent distribution function (TDF). For the TDF, we formulate it as a mixture of von Mises–Fisher (vMF) distributions within a directional statistics framework [4].

We present results across various settings. Under orthographic view, where parallax effects are absent, our results exhibit negligible deviation from the reference. Under perspective view, our results remain closely aligned with the reference and achieve real-time rendering filtering.

Concretely, our main contributions are as follows:

- A joint filtering framework for BCSDFs and tangent maps, enabling real-time level-of-detail (LoD) rendering of BCSDFs.
- An analytical effective BCSDF formulation based on the von Mises–Fisher (vMF) distribution, preserving intrinsic optical scattering properties while admitting closed-form convolution with TDFs.
- A novel Clustered Control Variates (CCV) integration scheme for efficiently approximating the convolution of the TT lobe.

## 2. Related Work

**Bidirectional curve-scattering distribution function (BCSDF).** Rendering curve-based structures such as hair or cloth necessitates modeling them as cylindrical fiber assemblies. The foundational physics-based hair scattering model by Marschner et al. [9] formulates light transport through three distinct scattering paths (R, TT, and TRT lobes). This framework relies on geometrically smooth cylindrical fiber assumptions. d’Eon et al. [10] extended the Marschner model to account for azimuthal roughness. While these far-field models capture macroscopic appearance, Chiang et al. [11] demonstrated superior physical accuracy using near-field formulations that resolve local light–fiber interactions. Yuksel et al. [12] decomposed global illumination into dual scattering components: volumetric diffusion and localized fiber interactions. Khungurn et al. [13] extended the BCSDF model, incorporating only the R and TT lobes. To approximate multiple scattering, modern fiber-level models introduce an empirical diffuse lobe (D lobe) [14]. Given the extreme fiber densities in hair/cloth structures, recent research has transitioned to aggregate representations [15,16,17], where fiber bundles are treated as volumetric ply/yarn structures for efficient rendering. Wu and Yuksel [18] simplified the geometry by estimating the number of fibers within each yarn, enabling efficient computation. More recent studies [19,20] have demonstrated the potential of neural networks to be applied to such fabric material models, offering a promising direction for future advancements.

**BRDF–Normal Filtering.** Normal map filtering persists as a fundamental challenge in real-time rendering. The seminal work by Fournier [21] pioneered the use of multiple-lobe Phong fits for normal distribution approximation. Subsequent work by Becker et al. [22] enabled smooth LoD transitions through trilinear interpolation over precomputed reflectance volumes. Toksvig et al. [7] introduced real-time normal map mipmapping via variance-preserving normal distribution filtering. Han et al. [6] formulated a theoretical framework using spherical harmonics (SH) and von Mises–Fisher (vMF) mixtures, although it requires iterative nonlinear optimization incompatible with real-time constraints. In contrast, LEAN mapping [5] and LEADR mapping [23] utilize Gaussian statistical moments (the first and second moments of surface normals) to enable memory-efficient linear filtering. Recent work by Xu et al. [4] proposes the joint filtering of MIP-mapped BRDFs and normal maps, while Xavier et al. [24] introduced a frequency-aware reconstruction framework for preserving high-frequency specular lobes. We recommend this survey [25] for a comprehensive review.

**Control Variates in Rendering.** Control variates represent a foundational variance reduction methodology for integral estimation problems. The technique operates by decomposing the integrand into an analytically tractable base term and a residual term. While the residual term is estimated via Monte Carlo sampling, this framework achieves significantly reduced variance when the base component closely approximates the original integrand. This approach has been successfully applied in rendering systems for direct illumination integrals with visibility constraints [26,27].

## 3. Method

Our framework computes effective BCSDFs through analytic convolution without requiring precomputation. Section 3.1 establishes theoretical foundations. Section 3.2 presents linear combinations of discrete BCSDFs via von Mises–Fisher (vMF) distribution approximations. Section 3.3 derives analytic solutions for the effective BCSDF, defined as the convolution of the tangent distribution function (TDF) with the proposed BCSDF. Implementation details are provided in Section 3.4.

### 3.1. Background

Before delving into our method, we first introduce the conventional formulations of the BCSDF, the TDF, and the effective BCSDF from previous work.

**BCSDFs.** First introduced by Marschner et al. [9], through the approximation of fibers as transparent circular cylinders, this model effectively captures microscale geometric details. Building upon this framework, we employ the fiber-based BCSDF formulation proposed by Zhu et al. [28], which disregards local scattering effects and consists of three lobes: reflection (R), transmission (TT), and diffuse (D). Its BCSDF is given by

(1) $$f\left(\theta_{i},\theta_{r},\phi_{i},\phi_{r}\right)=\sum_{p\in \{R,TT,D\}}a_{p}\cdot M_{p}\left(\theta_{h}\right)\cdot N_{p}\left(\phi \right)/\cos^{2}\theta_{i}$$

where $\theta_{h}$ is the longitudinal half-angle, defined as $\theta_{h}=\left(\theta_{r}+\theta_{i}\right)/2$, and $\phi =(\phi_{i}-\phi_{r})$ is the relative exiting azimuth. The terms $M_{p}$ and $N_{p}$ denote the longitudinal scattering function and the azimuthal scattering function, respectively, as follows:

(2) $$\begin{array}{cccc} M_{R}\left(\theta_{h}\right) & =g(\beta_{R}^{M};\theta_{h}), & N_{R}\left(\phi \right) & =\frac{1}{2\pi }\\ M_{TT}\left(\theta_{h}\right) & =g(\beta_{TT}^{M};\theta_{h}), & N_{TT}\left(\phi \right) & =g(\beta^{N},\phi )\\ M_{D} & =\frac{1}{\pi }, & N_{D} & =\frac{1}{2\pi } \end{array}$$

where $\beta^{M}$ and $\beta^{N}$ represent the longitudinal and azimuthal roughness.

**TDF.** Following previous work [29], we represent the TDF γ using a vMF distribution defined by a mean vector $\mu_{t}$ and a concentration factor $\kappa_{t}$ as:

(3) $$\gamma \left(t;\mu_{t},\kappa_{t}\right)=\frac{\kappa_{t}}{2\pi }e^{\kappa_{t}\left(\mu_{t}\cdot t-1\right)}$$

As demonstrated in prior work [4], a vMF distribution parameterized by μ and κ can be equivalently represented using an unnormalized vector $r\in R^{3}$, with further derivations provided in [29].

(4) $$\frac{r}{\left||r|\right|}=\mu ,\;\frac{3||r||-{\left||r|\right|}^{3}}{1-{\left||r|\right|}^{2}}=\kappa$$

The TDF mixture can thus be formulated as a linear combination of vMF parameter vectors r, analogous to approximating the average of multiple vMF distributions with a single vMF distribution.

(5) $$r_{avg}=\frac{1}{N}\sum_{i=1}^{N}r^{\left(i\right)}$$

**Effective BCSDF.** The appearance of objects at large scale can be described using patch-wise effective BCSDF $f_{\mathrm{eff}}\left(\Omega ,\omega_{i},\omega_{r}\right)$:

(6) $$f_{\mathrm{eff}}\left(\Omega ,\omega_{i},\omega_{r}\right)=\frac{1}{\left|\Omega \right|}\int_{\Omega }f\left(x,\omega_{i},\omega_{r}\right)dx$$

where Ω is a pixel footprint, representing a small neighborhood around a given surface point and |Ω| denotes the surface area of Ω. We discretize Equation (6) by assuming that the pixel footprint Ω contains N discrete texels with BCSDFs $f_{\mathrm{tex}}^{\left(1\right)},f_{\mathrm{tex}}^{\left(2\right)},\ldots ,f_{\mathrm{tex}}^{\left(N\right)}$:

(7) $$f_{\mathrm{eff}}\left(\Omega ,\omega_{i},\omega_{r}\right)=\frac{1}{N}\sum_{i=1}^{N}f_{\mathrm{tex}}^{\left(i\right)}\left(\omega_{i},\omega_{r}\right)$$

### 3.2. BCSDF Filtering via vMFs

As established in the preceding analysis, the effective BCSDF is formulated as the ensemble average of discrete texels (Figure 1). We propose parameterizing $f_{\mathrm{tex}}$ using a vector $\omega_{h}$, enabling its representation via vMF distributions. Following directional statistics theory [4], the average of multiple vMF distributions converges to a single vMF distribution—a critical property enabling analytic solutions.

The angular parameters $\theta_{h}$ and ϕ, which define the BCSDF, are consolidated into a vector $\omega_{h}=\left(\sin\theta_{h}\cos\phi ,\sin\theta_{h}\sin\phi ,\cos\theta_{h}\right)$, yielding the vMF-approximated BCSDF $f_{\mathrm{tex}}$:

(8) $$f_{\mathrm{tex}}\approx \frac{\kappa }{2\pi }e^{\kappa (t\cdot \omega_{h}-1)}$$

For the three lobes of the BCSDF, their respective formulations are reparameterized by $\omega_{h}$ as follows:

**D Lobe.** Both the longitudinal and azimuthal functions in the D term are defined as constants in Equation (2), requiring no specialized treatment.

**R Lobe.** For the longitudinal function $M_{R}$ in Equation (2), we present a new representation. Based on the definition of $\theta_{i}$ and $\theta_{r}$ in $M_{R}$, which represent the angles between the incident/outgoing light and the normal plane, these angles are equivalently redefined as angular deviations from the tangent vector. Building upon prior work [30], we derive a mathematical connection between Gaussian functions and vector inner products, as formalized in Equation (8). To leverage this formulation, we further develop a novel approximation of the $M_{R}$ term:

(9) $$M_{R}^{\prime }=\frac{s+1}{2\pi }{\left(\cos\theta_{h}\right)}^{s}$$

where $\cos\theta_{h}$ can be approximated with $\omega_{h}$ and t:

(10) $$\cos\theta_{h}\approx 1-\omega_{h}\cdot t,\;\left(\theta_{h}=\frac{\pi }{2}-\arccos\left(\omega_{h}\cdot t\right)\right)$$

**Figure 2:**
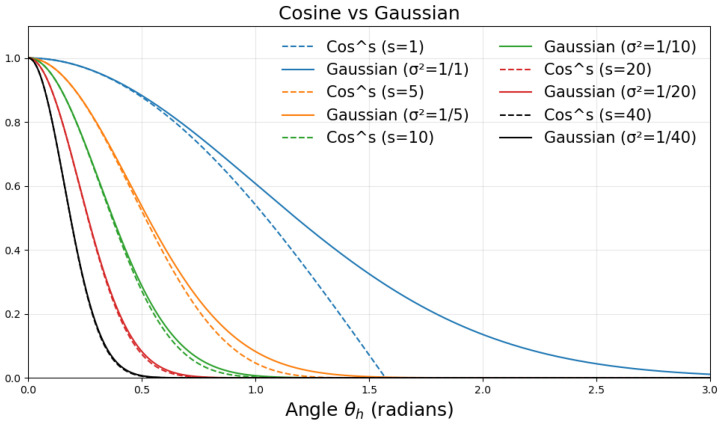
A comparison of the cosine term and Gaussian highlight shape as a function of angle $\theta_{h}$. Our form approximates a Gaussian as the specular exponent s increases.

Figure 2 establishes the similarity between the cosine formulation in Equation (9) and conventional Gaussian.

**TT Lobe.** While the longitudinal component $M_{TT}$ necessitates similar treatment to $M_{R}$, the azimuthal term $N_{TT}$ presents two fundamental challenges: first, the difficulty in representing it using von Mises–Fisher (vMF) distributions, and second, the mathematical intractability of expressing its convolution with the longitudinal function using vMF [31]. We address these challenges through our Clustered Control Variates (CCV) technique detailed in Section 3.3.

### 3.3. Closed Form of vMF Convolution

Solving Equation (6) conventionally relies on Monte Carlo methods employing extensive sampling operations. To address this problem, the previous method typically requires a large volume of commonly precomputed data, which imposes significant pressure on GPU memory. Our analytic solution overcomes these limitations through expressing the effective BCSDF as the convolution of two vMF distributions.

Reformulating Equation (6) into continuous form [6]:

(11) $$f_{\mathrm{eff}}\left(\omega_{h}\right)=\int_{S^{2}}f_{\mathrm{tex}}\left(t,\omega_{h}\right)\gamma \left(t\right)dt$$

The integration over discrete points on domain Ω has been reformulated through a parameterization of $t\in S^{2}$, where t denotes the tangent direction and γ(t) represents TDF. Critically, γ(t) becomes mathematically equivalent to a summation of Dirac delta distributions on the sphere, explicitly encoding fine-scale tangent orientations. As established in the background (Section 3.1), we define the TDF as a mixture of vMF distributions, where each fine-scale tangent corresponds to a narrow vMF lobe.

We employ a three-dimensional frequency domain analysis by expanding Equation (11) using spherical harmonic (SH) basis functions $Y_{lm}\left(\cdot \right)$. This approach is analogous to Fourier series decomposition in 2D frequency domain analysis, except that the spectral transformation is performed on the unit sphere. Here, the index *l* denotes the order of the spherical harmonic (satisfying l≥0), while *m* represents the azimuthal component with the constraint −l≤m≤l.

(12) $$\begin{array}{cc} \gamma (t) & =\sum_{l=0}^{\infty }\gamma_{l}Y_{l0}\left(t\right)\\ f_{\mathrm{tex}}\left(\omega_{h}\cdot t\right) & =\sum_{l=0}^{\infty }f_{l}Y_{l0}\left(\omega_{h}\cdot t\right)\\ f^{\mathrm{eff}}\left(\omega_{h}\right) & =\sum_{l=0}^{\infty }f_{l}^{\mathrm{eff}}Y_{l0}\left(\omega_{h}\right) \end{array}$$

This formulation is analogous to the standard functional expansion of Fourier series. Owing to the radial symmetry of the expanded function, the spherical harmonics reduce to Zonal Harmonics (ZH) $Y_{l0}\left(\cdot \right)\left(m=0\right)$, where the function depends solely on the polar angle θ and becomes independent of the azimuthal angle ϕ. Following prior work [3], the spherical harmonic coefficients in this configuration admit a simple product formula, analogous to how standard convolutions can be expressed as products of Fourier coefficients.

(13) $$\begin{array}{cc} f_{l}^{\mathrm{eff}} & =\sqrt{\frac{4\pi }{2l+1}}f_{l}\gamma_{l}\\ \; & ={\hat{f}}_{l}\gamma_{l},\;with\;{\hat{f}}_{l}=\sqrt{\frac{4\pi }{2l+1}}f_{l} \end{array}$$

Next, we proceed to derive analytical expressions for each lobe of the BCSDF individually through frequency-domain analysis.

**D Lobe** The D lobe is all constant; no need to calculate the convolution.

**R Lobe** The R lobe is a constant multiple of the longitudinal function $M_{R}$ with the form of Equation (9). By substituting Equations (3), (9), and (10) into Equation (11), we obtain

(14) $$\begin{array}{cc} f_{\mathrm{eff}}^{R}\left(\omega_{h}\right) & =\int_{S^{2}}f_{\mathrm{tex}}^{R}\left(t,\omega_{h}\right)\gamma \left(t\right)dt\\ \; & =\int_{S^{2}}M_{R}^{\prime }\left(t,\omega_{h}\right)N_{R}\left(t,\omega_{h}\right)\gamma \left(t\right)dt\\ \; & =\frac{1}{2\pi }\int_{S^{2}}M_{R}^{\prime }\left(t,\omega_{h}\right)\gamma \left(t\right)dt \end{array}$$

According to previous work [3], the spherical harmonic coefficients of $M_{R}^{\prime }$ are ${\hat{f}}_{l}\approx e^{-l^{2}\;/2s}$. Let $\Lambda_{l}=\sqrt{\frac{4\pi }{2l+1}}$; we have $\Lambda_{l}\gamma_{l}=e^{-l^{2}\;/2\kappa_{t}}$ [6]. Substituting these into Equation (13), the result after convolution has the same form [4] with the reflection term $M_{R}^{\prime }$:

(15) $$\begin{array}{cc} \Lambda f_{l}^{\mathrm{eff}} & ={\hat{f}}_{l}\Lambda_{l}\gamma_{l}\\ \; & =e^{-l^{2}\;/2s}e^{-l^{2}\;/2\kappa_{t}}\\ \; & =e^{-l^{2}\;/2s^{\prime }}\\ ⟹f_{\mathrm{eff}}^{R}\left(\omega_{h}\right) & =\frac{1}{2\pi }\cdot \frac{\kappa }{2\pi }e^{\kappa (\cos\theta_{h}-1)} \end{array}$$

where $\kappa =s^{\prime }+1$, $s^{\prime }=\kappa_{t}\cdot s/\left(\kappa_{t}+s\right)$.

**TT Lobe** Since only the TT lobe’s longitudinal function is modified, the solution for $f_{\mathrm{eff}}^{TT}$ requires excessive per-pixel samples for noise-free results. To address this computational challenge, we employ Clustered Control Variates (CCV) [2], extending prior methodologies. To avoid brute-force Monte Carlo sampling for the residual term, we leverage structural correlations by partitioning the footprint into clusters with internal similarity. This decomposition is formulated as:

(16) $$\begin{array}{cc} f_{\mathrm{eff}}^{TT}\left(\omega_{h}\right) & =\int_{S^{2}}M_{TT}^{\prime }\left(t,\omega_{h}\right)N_{TT}\left(t,\omega_{h}\right)\gamma \left(t\right)dt\\ \; & =\int_{\Omega_{j}}\left(M_{TT}^{\prime }\left(t,\omega_{h}\right)N_{TT}\left(t,\omega_{h}\right)-\bar{N}\right)\gamma \left(t\right)dt+\sum_{j=1}^{K}\bar{N_{j}}M_{TT}^{j} \end{array}$$

**Figure 3:**
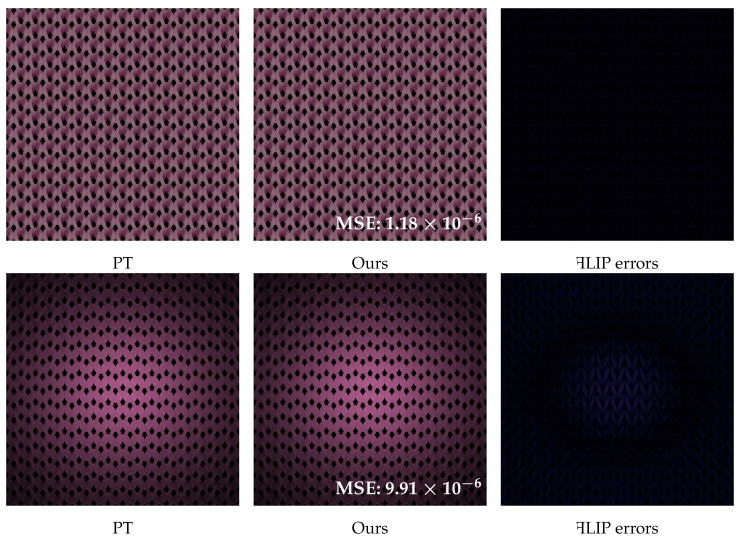
The result of our effective BCSDF; the first line is the result of R+D lobe and the second line is the TT lobe (only used base term). Comparative ꟻLIP error [32] analysis with path tracing (using full yarn geometry) under the parallax-free setting demonstrates that our method achieves near-reference accuracy, with MSE values of $1.18\times 10^{-6}$ (R+D) and $9.91\times 10^{-6}$ (TT).

The summation component corresponds to the base term, while the integral corresponds to the residual term, where $\Omega_{j}$ denotes the texels of cluster *j* in the pixel footprint Ω. Here, $\bar{N_{j}}$ indicates the average azimuthal function value over cluster $\Omega_{j}$. As shown in Figure 3, our experiments demonstrate that the residual term becomes negligible under an optimized cluster strategy, yielding the approximation $f_{\mathrm{eff}}^{TT}\approx \sum_{j}^{K}\bar{N_{j}}M_{TT}^{j}$. The cluster-partitioning methodology is detailed in Section 3.4.

### 3.4. Implementation

We now detail our implementation. The core of our framework employs mixtures of vMF distributions. While previous methods rely on the computationally expensive vMF-mixture fitting, which is impractical for real-time rendering, we adopt the angular-domain partitioning approach proposed by Xu et al. [4]. This divides the spherical domain $S^{2}$ into *K* clusters, each aggregating directions with similar vMF eigenvector orientations r, thereby eliminating the need for explicit fitting. Our implementation then performs linear filtering of vMF distributions within each cluster.

Our method builds upon the mixture modeling of tangent distributions. The pipeline begins with a tangent map as input. First, we partition the tangent directions into *K* clusters, each grouping orientations with similar directional vectors. Building upon the normal filtering framework established in previous work [4], and given that our tangent vectors are defined over the entire sphere, we adpot the setting K=16. Subsequently, we construct a MIP-mapped texture array that stores cluster assignments through downsampling operations to generate multi-level representations. This configuration achieves optimal trade-offs between memory efficiency and visual fidelity for full spherical domain coverage, determined through empirical validation.

As analytically established in Section 3.3, the effective TT lobe $f_{\mathrm{eff}}^{TT}$ reduces to the base term exclusively under angular clustering. The formulation derives $\bar{N_{j}}$ using the vMF lobe center vector (serving as clustered tangent direction), incorporating the $\cos\theta_{i}$ term from Equation (1) accordingly.

The per-pixel shading operates through three computational stages: First, determine the appropriate MIP-map level through standard texture sampling. Next, fetch the cluster vector r and solve for the tangent direction $\mu_{t}$ and concentration parameter κ via Equation (4). Finally, integrating cluster-wise contributions to compute the effective BCSDF.

## 4. Result

The proposed method is implemented in Vulkan 1.2 and evaluated on a standard PC equipped with a Intel® Xeon(R) W-2123 CPU @ 3.60GHz × 8 CPU and an NVIDIA GeForce RTX 2070 GPU. Our approach is a surface-based method that accepts two inputs: a base mesh and a tangent map that encodes the knit pattern (with a resolution of 256, resulting in negligible memory usage). The reference implementation is constructed by projecting yarn control points onto a geometric plane to reconstruct the full yarn-level microstructure, which is then rendered using path tracing with 1024 samples per pixel (spp) to provide high-fidelity ground truth. For the comparison methods, Toksvig. [7] and Zirr. [8], we apply their respective filtering algorithms to the tangent–space representation to obtain comparable results.

Both the proposed and comparison methods are evaluated using a combination of qualitative inspection and quantitative metrics, including ꟻLIP error [32] (with bright yellow corresponding to the maximum error and dark representing the minimum error) and mean squared error (MSE), to rigorously assess the performance. All quantitative results are summarized in Table 1 for clarity. Since the proposed and comparison methods rely on tangent map-based techniques, memory usage is negligible.

**Figure 4:**
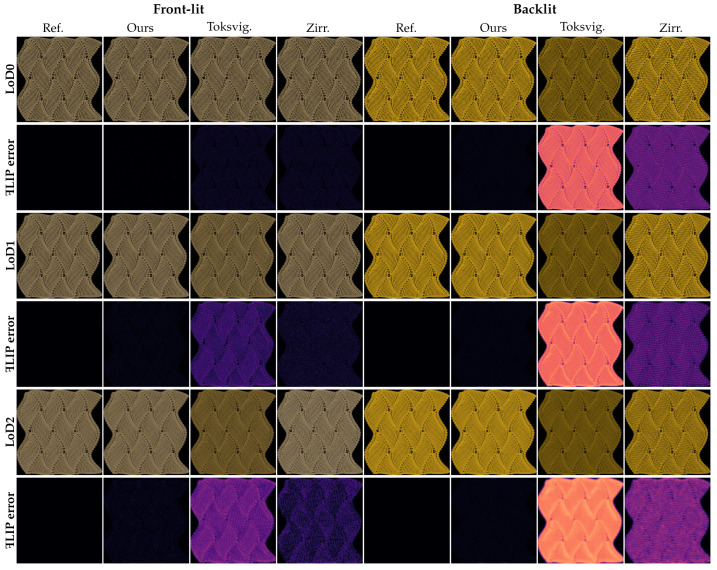
Planar knitted patterns were rendered under frontlight (R+D lobe) and backlight (TT+D lobe) conditions at various levels of detail (LoD) using orthographic projection. Each row begins with rotated labels on the left for identification. Compared with the path-traced reference using full yarn geometry (Ref.), our approach employs the effective BCSDF (Ours), while the comparison methods include mipmapped tangent maps (Toksvig.) and bi-tangent convolution (Zirr.). Across all scenarios, our method consistently exhibits negligible ꟻLIP error relative to the reference, significantly outperforming the comparison methods.

**Figure 5:**
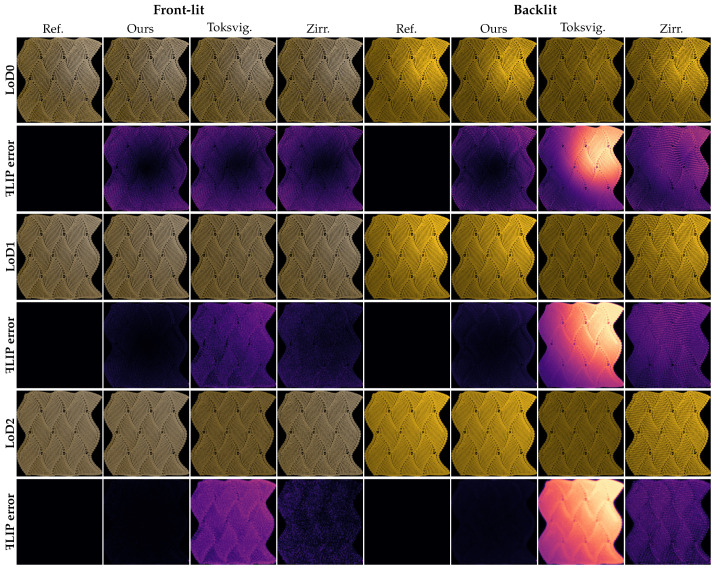
Planar knitted patterns were rendered under frontlight (R+D lobe) and backlight (TT+D lobe) conditions at multiple levels of detail (LoD) using perspective projection. Each row is labeled on the left with rotated identifiers for ease of reference. Images at higher LoD levels are presented with magnification for comparative analysis. When compared to the path-traced reference (Ref.), our approach (Ours), mipmapped tangent maps (Toksvig.), and bi-tangent convolution (Zirr.), the ꟻLIP error indicates that our method consistently reduces error as LoD increases, in stark contrast to the comparison methods, where errors accumulate at higher LoD levels.

*Planar knitted pattern.* Surface-based methods remain fundamentally limited by their inability to capture parallax effects, particularly critical at grazing incidence angles. Therefore, we validate the accuracy of our method through dual validation frameworks; Figure 4 and Figure 5, respectively, present the results for planar knitted pattern under orthographic and perspective projections. Apart from the camera projection settings, all other scene parameters remain identical.

Under *orthographic projection*, as shown in Figure 4, the differences between our method and the reference are nearly negligible across all LoD levels, for both front and back lighting conditions. The MSE values listed in Table 1 consistently exhibit low error levels across all evaluated scenes. In contrast, the comparison methods exhibit MSE values that are generally 2 to 3 orders of magnitude higher than ours, and their ꟻLIP errors increase significantly with increasing LoD level. This is primarily because, at higher LoD levels, the number of subpixel-scale scattering events accumulates within each pixel footprint. Traditional filtering schemes are incapable of accurately capturing such dense and anisotropic scattering, especially under backlit scenarios. In contrast, our effective BCSDF leverages the clustering strategy that enables more accurate modeling of the transmitted specular highlights associated with the TT lobe.

Since orthographic projection eliminates parallax effects, this experiment provides strong evidence for the correctness and robustness of our method across varying viewing distances in parallax-free settings.

For the *perspective projection* case, as shown in Figure 5, our method consistently outperforms the comparison methods in both qualitative and quantitative evaluations. The ꟻLIP error maps demonstrate a clear trend: as the LoD level increases, the error in our method decreases, while the errors in the comparison methods progressively accumulate. Similarly, our MSE results, shown in Table 1, are consistently lower than those of the baselines, particularly under backlit conditions, where our method achieves at least an order-of-magnitude lower MSE. This improvement stems from our joint filtering strategy that convolves the BCSDF with the tangent distribution, rather than treating tangent filtering in isolation as in the comparison methods.

The combined results in Figure 4 and Figure 5 also highlight that the primary discrepancies in our method arise from parallax effects. The downward trend in error with increasing LoD in Figure 5 further supports this conclusion: as the LoD increases, the distance between the geometry and the camera grows, reducing the influence of grazing-view parallax. This dual validation under orthographic and perspective projections confirms that our effective BCSDF model robustly handles the accumulation of complex scattering behavior within a single-pixel footprint. The residual differences from the reference are largely attributable to parallax—a known limitation of surface-based methods and outside the scope of this work.

**Figure 6:**
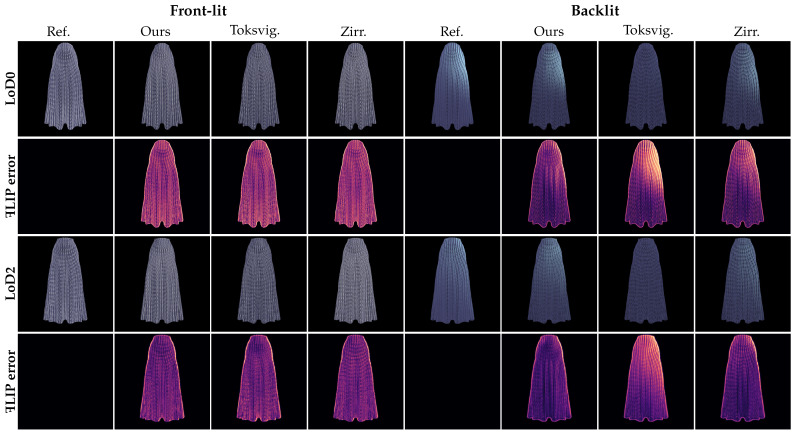
A comparison of rayon dress renderings with knit stitches under both a frontlight and backlight, generated via path tracing (full yarn geometry; Ref.), for our method, Toksvig’s approach, and Zirr’s approach. Images at LoD2 level are presented with magnification for comparative analysis.

*Real cloth scene.*Figure 6 illustrates our knitted stitch tiling on a dress. The ꟻLIP error map and MSE values in Table 1 indicate that our method outperforms the comparison methods of Toksvig et al. [7] and Zirr et al. [8], as they fail to handle the blending of multiple tangent directions within a single pixel. In backlight conditions, where conventional methods struggle to properly convolve the TT lobe with the TDF, our approach achieves a visual fidelity closer to the reference result in handling transmitted specular highlights. The quantitative analysis from Table 1 shows that our method reduces MSE compared to other methods, and for backlight scenarios, the MSE of our approach is improved by at least 25%.

**Figure 7:**
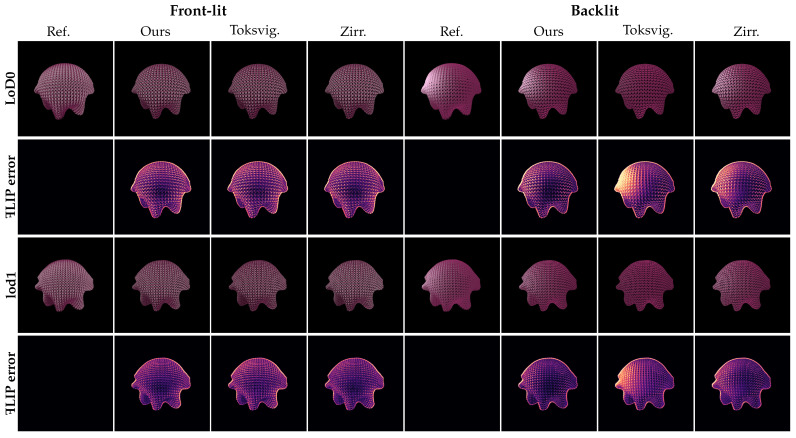
Knitted silk tablecloth rendering results under both light settings.

*Knitted Tablecloth.* In Figure 7, we present a real knitted silk tablecloth scenario under varying lighting conditions, including both front- and backlit setups. Under the front-lit condition, the primary difference between our method and the comparative approaches lies in the handling of the edges of overlapping yarns, as our method better handles the aggregation of multiple tangents within pixels. For the backlit scenario, our clustered control variates strategy for the TT lobe leads to improved results in both visual quality and quantitative metrics, outperforming the comparative approaches.

## 5. Conclusions

This paper introduces a joint linear MIP-mapping technique for BCSDFs integrated with tangent maps, enabling the efficient computation of effective BCSDF. We represent both BCSDF and tangent distributions using von Mises–Fisher(vMF) formulations, leading to closed-form solutions for effective BCSDF derivation. Our work reformulates the conventional BCSDF into vMF-compatible representations without the need for an expensive fitting method, achieved through discrete cluster splitting during linear vMF filtering. For the TT lobe in BCSDF, we use the Clustered Control Variates scheme to obtain an approximation.

The effectiveness of our algorithm has been validated through multiple experimental scenarios. The proposed method can compute the equivalent BCSDF efficiently while maintaining real-time rendering rates. Under parallax-free conditions, the MSE of our method outperforms the comparative approaches by at least one order of magnitude. In real rendering scenarios with parallax, the MSE of our method shows an improvement of at least 25% in backlit conditions compared to the competing algorithms, outperforming all comparison methods.

## 6. Future Work

Our method still has some limitations that remain for future work. First, we have not handled the parallax effects—a critical component for photorealistic rendering at near-grazing angles. While numerous existing methods address parallax, their seamless integration with our framework remains a significant challenge. Second, we would also like to extend our method to support the multiple scattering of BCSDFs, aiming for more realistic BCSDF material rendering results.

## Figures and Tables

**Table 1:** MSE quantitative data for all experimental scenarios.

Scenes	LoDs	Methods	Frontlight	Backlight	Time *
planar pattern (orthographic)	LoD0	Ours	$1.02\times 10^{-6}$	$3.95\times 10^{-6}$	3.27 ms
		Toksvig.	$4.64\times 10^{-4}$	$1.54\times 10^{-2}$	2.78 ms
		Zirr.	$4.66\times 10^{-4}$	$2.43\times 10^{-3}$	2.93 ms
	LoD1	Ours	$2.29\times 10^{-5}$	$8.72\times 10^{-6}$	2.56 ms
		Toksvig.	$9.44\times 10^{-4}$	$5.26\times 10^{-3}$	2.38 ms
		Zirr.	$3.85\times 10^{-4}$	$6.97\times 10^{-4}$	2.42 ms
	LoD2	Ours	$9.01\times 10^{-6}$	$2.31\times 10^{-6}$	1.93 ms
		Toksvig.	$6.04\times 10^{-4}$	$1.69\times 10^{-3}$	1.72 ms
		Zirr.	$1.64\times 10^{-4}$	$3.74\times 10^{-4}$	1.82 ms
planar pattern (perspective)	LoD0	Ours	$5.67\times 10^{-3}$	$3.20\times 10^{-3}$	3.21 ms
		Toksvig.	$6.63\times 10^{-3}$	$1.44\times 10^{-2}$	2.75 ms
		Zirr.	$6.60\times 10^{-3}$	$4.46\times 10^{-3}$	2.91 ms
	LoD1	Ours	$1.41\times 10^{-4}$	$6.64\times 10^{-5}$	2.57 ms
		Toksvig.	$1.49\times 10^{-3}$	$5.49\times 10^{-3}$	2.32 ms
		Zirr.	$8.23\times 10^{-4}$	$8.97\times 10^{-4}$	2.40 ms
	LoD2	Ours	$3.88\times 10^{-6}$	$3.18\times 10^{-6}$	1.87 ms
		Toksvig.	$6.72\times 10^{-4}$	$1.80\times 10^{-3}$	1.70 ms
		Zirr.	$2.20\times 10^{-4}$	$2.58\times 10^{-4}$	1.77 ms
dress	LoD0	Ours	$1.52\times 10^{-2}$	$7.34\times 10^{-3}$	2.22 ms
		Toksvig.	$1.74\times 10^{-2}$	$1.17\times 10^{-2}$	1.92 ms
		Zirr.	$1.75\times 10^{-2}$	$9.95\times 10^{-3}$	2.12 ms
	LoD2	Ours	$3.06\times 10^{-3}$	$1.33\times 10^{-3}$	0.92 ms
		Toksvig.	$3.88\times 10^{-3}$	$2.34\times 10^{-3}$	0.79 ms
		Zirr.	$3.72\times 10^{-3}$	$1.85\times 10^{-3}$	0.87 ms
table cloth	LoD0	Ours	$8.61\times 10^{-3}$	$8.54\times 10^{-3}$	1.80 ms
		Toksvig.	$9.78\times 10^{-3}$	$1.35\times 10^{-2}$	1.62 ms
		Zirr.	$9.93\times 10^{-3}$	$1.09\times 10^{-2}$	1.71 ms
	LoD1	Ours	$2.06\times 10^{-3}$	$1.63\times 10^{-3}$	1.17 ms
		Toksvig.	$2.59\times 10^{-3}$	$2.56\times 10^{-3}$	1.08 ms
		Zirr.	$2.31\times 10^{-3}$	$2.13\times 10^{-3}$	1.11 ms

* Time refers to the average rendering time per frame. All rendered results were output at a resolution of 1K.

## Data Availability

The data presented in this study are available on request from the corresponding author.

## Abbreviations

The following abbreviations are used in this manuscript:

BCSDF	Bidirectional Curve Scattering Distribution Function
TDF	Tangent Distribution Function
vMF	von Mises–Fisher Distribution
CCV	Clustered Control Variates
MSE	Mean squared error
